# Association between dyslipidemia and blood lipids concentration with smoking habits in the Kurdish population of Iran

**DOI:** 10.1186/s12889-020-08809-z

**Published:** 2020-05-13

**Authors:** Mehdi Moradinazar, Yahya Pasdar, Farid Najafi, Soodeh Shahsavari, Ebrahim Shakiba, Behrooz Hamzeh, Negin Fakhri

**Affiliations:** 1 Behavioral Disease Research Center, Kermanshah University of Medical Sciences, Kermanshah, Iran; 2 Research Center for Environmental Determinants of Health, School of Public Health, Kermanshah University of Medical Sciences, Kermanshah, Iran; 3 Nutritional Sciences Department, School of Public Health, Kermanshah University of Medical Sciences, Kermanshah, Iran; 4 Behavioral Disease Research Center, Kermanshah University of Medical Sciences, Kermanshah, Iran; 5 Student’s research committee, Faculty of Health, Kermanshah University of medical sciences, Kermanshah, Iran

**Keywords:** Dyslipidemia, Smoking, Blood lipids, Current smoker, Former smoker

## Abstract

**Background:**

Smoking is the most preventable cause of most chronic diseases such as cardiovascular disease (CVD). Dyslipidemia is also an important risk factor for CVD. Yet, research has provided contradicting findings regarding the association between smoking and blood lipids. This paper examines the relationship between dyslipidemia and smoking based on the results of a cross-sectional sample of a Kurdish population in western Iran.

**Methods:**

This population-based study was derived from the recruitment phase of Ravansar Non-Communicable Disease (RaNCD) cohort study. Logistic regression model adjusted by confounding variables was used to determine the relationship between smoking and blood lipid components. In addition, dose-response relationship between blood lipids and the number of smoked cigarettes was evaluated.

**Results:**

For the purpose of this study, 7586 participants were examined. The lifetime prevalence of smoking was 19.9%, and 11.8% were current smokers. The prevalence of dyslipidemia in current smokers (54.9%) was higher than former smokers (43.9%) and in turn former smokers higher than non-smokers (38.0%). Current smokers had greater risk of abnormal HDL cholesterol [OR (95% CI), 2.28(1.98 -2.62)] and triglyceride [OR (95% CI), 1.37(1.15 -1.67)] compared to non-smokers. There was no significant difference in total cholesterol and LDL cholesterol between the two groups. A dose-response relationship was found between the number of cigarettes smoked and HDL-C and TG but no relationship was observed in terms of total cholesterol and LDL-C.

**Conclusions:**

As compared to non-smokers, current smokers and former smokers had abnormal HDL-C and triglyceride and abnormal total cholesterol and triglyceride, respectively. After quitting smoking, heavy smokers showed a more normal HDL-C and total cholesterol levels than the people who tended to smoke a lower number of cigarettes per day.

## Background

There is strong evidence that dyslipidemia increases the risk of cardiovascular diseases [1, 2]. It accounts for more than half of the deaths in different societies [3]. Due to the effect it has on the cardiovascular system, the metabolism of fat in the human body is significant [4, 5]. Abnormality in each component of the blood lipids results in the development of chronic non-communicable diseases [6]. In addition to the association between the prevalence of dyslipidemia with ethnicity and, social, economic and cultural characteristics of populations, its determinants (life style) are also varied among different societies.

Although several major factors have already been identified for the occurrence of dyslipidemia, other unknown risk factors also exist [7–12]. Insufficient knowledge of dyslipidemia has resulted in inappropriate planning and employment of ineffective treatment methods. Factors such as age, body mass index, alcohol consumption, and lifestyle are known as risk factors for dyslipidemia [13].

Smoking is believed to change the level of blood lipids. Despite the fact that there is no definite relationship between smoking and blood lipids [5, 14, 15], some studies have shown that cigarette smoking is likely to alter blood lipid levels in the serum through the absorption of nicotine which changes the mechanism of blood lipids [16]. Some studies report that nicotine increases triglyceride, total cholesterol, LDL cholesterol (LDL-C), and decreases HDL cholesterol (HDL-C). Other studies show that smoking reduces HDL-C, LDL-C and total cholesterol and increases triglyceride [17, 18].

Apart from its relationship to lung cancer and heart diseases, smoking is also associated with many non-communicable chronic health problems [19]. In general, a number of health conditions are associated with tobacco use due to its effect on the physical condition and immune system of the smokers. Nevertheless, cigarette smoking is a factor that can be controlled easily through implementing preventive and educational programs based on research on how it alters blood lipids. Given that, there has been no evidence of a unanimous association between dyslipidemia and smoking [5, 14, 15]. Therefore, we examined the relationship between smoking and blood lipids in the largest population-based study in western Iran.

## Methods

### The study population

This study was derived from the recruitment phase of Ravansar Non-Communicable Disease (RaNCD) cohort study in the Kurdish population of western Iran. The recruitment phase began in November 2014 and ended in February 2017 through which participants who had met the criteria were selected to participate in the study. A total number of 10065 subjects willingly participated and signed the written informed consent letter. For further details refer to the protocol and research guide [20, 21].

### Inclusion and exclusion criteria

Inclusion criteria were residency, being in the age range of 35-65, living in the area for at least one year (living in that city for at least 9 months), willingness to participate and complete the research, providing signed written informed consent letter, and ability to communicate with the research team. In order to eliminate the effect of confounding variables, subjects with hepatitis (14 cases), diabetes (1008 cases), renal failure (101) and high blood pressure (1681), as well as those on medications for dyslipidemia (407 people) were excluded from the study (2479 subjects were excluded).

### Definition and measurements

For the purpose of this study, dyslipidemia was defined as LDL-C ≥160 mg/dL and /or total cholesterol ≥240 mg/dL and/or HDL-C<40 mg/dL and/or triglycerides >200 mg/dL [5]. The smoking habit assessment was conducted based on National Health Insurance Scheme (NHIS). It was defined in terms of the number of cigarettes and duration of smoking. The subjects were classified into three groups of smokers, non-smokers, and former smokers. Smokers were people who reported they had smoked at least 100 cigarettes, and they were currently smoking every day or every few days. The non-smoker group included those who reported they had not smoked at least 100 cigarettes during their lifetime. Former smokers were those who had quit with a history of smoking at least 100 cigarettes [22]. The number of smoked cigarettes referred to the number of cigarettes used on each day. Smoking habit was self-reported.

Socio-economic status (SES), the main variable indicative of the economic status of the family, was calculated by Principal Component Analysis (PCA) and considering the subjects’ economic and social variables. According to SES, the studied population was categorized into five quintiles: the poorest, the poor, the middle class, the rich, and the richest [23]. The anthropometric measurements were checked using an automated bioelectric impedance machine (In Body 770 BIOSPACE, Korea) with integrated automatic audiometer (BSM350) [24]. A 19-item inventory related to light, moderate and heavy physical activity was used to collect information about the subjects ‘physical activity. The metabolic equivalent of task (MET) of each activity was obtained based on Compendium of participant. Physical activity levels were classified as low (24-36.5 MET-hours per week), moderate (MET-36.6-44.9 hours per week) and heavy (MET-≥45 hours per week) [25]. To measure the quality of nutrition, Healthy Eating Index (HEI) – based on the 2015 guideline - was categorized into five groups. The Nutritional assessment was performed using the Food Frequency Questionnaire (FFQ) questionnaire [26].

### Statistical analysis

Data were described using the appropriate method (mean and standard deviation for quantitative variables and percentage for qualitative variable). The crude ORs with 95% confidence intervals within a forest plot were presented to examine the relationship between smoking and the risk of having abnormal blood lipids. The dose-response relationship between the number of cigarettes and blood lipids levels was evaluated without adjustment. To measure the relationship between smoking and blood lipids, a multiple logistic regression model (backward method) adjusted for the confounding variables was used. For all analyses, missing values were deleted (less than 1%). Stata version 14.2 and MetaXL software were used to collect data. All the tests were performed at a significance level of 0.05.

## Results

Of the 7586 subjects who were eligible to enter the study, 3715 (51.02%) were women, 6840 (90.17%) were married, and 1987 (26.19%) had poor physical activity (24-36.5 hours per week). The prevalence of dyslipidemia in smokers (54.9%) was higher than non-smokers (38.0%) and former smokers (43.9%).The prevalence of smoking was greater in men, married status, aged 56-65, low level of education, heavy physical activity, and normal BMI and BMI≤ 18.9 (Table 1).

**Table 1:** Baseline characteristics of non-smokers, former smokers, and current smokers

Variables		Total ***N***=7586 (N, %)	Non-smoker ***N***=6075 (N, %)	Former smoker ***N***=541 (N, %)	Current smoker ***N***=970 (N, %)	P
**Gender**	Male	3871(51.02)	2493(64.4)	466(12.04)	912(23.56)	<0.001
	Female	3715(48.98)	3582(96.42)	75(2.02)	58(1.56)	
**Age group (years)**	35-45	3941(51.95)	3369(85.49)	149(3.78)	423(10.73)	<0.001
	46-55	2448(32.27)	1855(75.78)	222(9.07)	371(15.16)	
	56-65	1197(15.77)	851(71.09)	170(14.2)	176(14.7)	
**Marital status**	Single	403(5.31)	373(92.56)	4(0.99)	26(6.45)	<0.001
	Married	6840(90.17)	5392(78.83)	525(7.68)	923(13.49)	
	Widow/ divorced	343(4.52)	310(90.38)	12(3.5)	21(6.12)	
**Educational status** (Years)	Illiterate	3064(40.39)	2523(82.34)	229(7.47)	312(10.18)	<0.001
	1-5	2146(28.29	1655(77.12)	152(7.08)	339(15.8)	
	6-9	880(11.60)	665(75.57)	53(6.02)	162(18.41)	
	10-12	849(11.19)	676(79.62)	67(7.89)	106(12.49)	
	13≥	647(8.53)	556(85.94)	40(6.18)	51(7.88)	
**Wealth index**	1st quintile ( the poorest)	1459(19.23)	1195(81.91)	89(6.10)	175(11.99)	0.664
	2nd quintile	1489(19.63)	1174(78.84)	114(7.66)	201(13.50)	
	3rd quintile	1489(19.63)	1177(79.05)	104(6.98)	208(13.97)	
	4th quintile	1533(20.21)	1212(79.06)	113(7.37)	208(13.57)	
	5th quintile (the richest)	1574(20.75)	1284(81.58)	118(7.50)	172(10.93)	
**Physical activity** (METs-***hours per week***)	24-36.5	1987(26.19)	1551(78.06)	146(7.35)	290(14.59)	<0.001
	36.6-44.9	3849(50.74)	3291(85.50)	220(5.72)	338(8.78)	
	≥45	1743(22.98)	1228(70.45)	175(10.04)	340(19.51)	
**BMI** kg/m2	≤18.5	149(1.96)	90(60.40)	10(6.71)	49(32.89)	<0.001
	18.6-24.9	2254(29.71)	1678(74.45)	173(7.68)	403(17.88)	
	25-29	3266(43.05)	2643(80.92)	241(7.38)	382(11.70)	
	30-34.9	1484(19.56)	1285(86.59)	95(6.40)	104(7.01)	
	>=35	383(5.05)	345(90.08)	17(4.44)	21(5.48)	
**Number of cigarette per day**	non- smoker	6075 (79.47)	-	-	-	0.089
	1-10	549(7.18)	-	189(41.18)	270(58.82)	
	10-20	272(3.55)	-	100(36.76)	172(63.24)	
	>20	748(9.84)	-	221(29.55)	*527(70.45)*	
**HEI**	1st quintile ( the unhealthy)	1587(20.92)	1247(78.58)	121(7.62)	219(13.8)	0.135
	2^nd^ quintile	1475(19.44)	1163(78.85)	100(6.78)	212(14.37)	
	3^rd^ quintile	1546(20.38)	1284(83.05)	93(6.02)	169(10.93)	
	4^th^ quintile	1699(22.40)	1361(80.11)	127(7.47)	211(12.42)	
	5th quintile (the healthy)	1279(16.86)	1020(79.75)	100(7.82)	159(12.43)	
**Abnormal HDL**	No	5226(68.89)	4357(83.37)	367(7.02)	502(9.61)	<0.001
	Yes	2306(30.40)	1677(72.72)	171(7.42)	458(19.86)	
**Abnormal LDL**	No	7397(97.51)	5924(80.09)	522(7.06)	951(12.86)	0.011
	Yes	135(1.78)	110(81.48)	16(11.85)	9(6.67)	
**Abnormal TG**	No	6467(85.25)	5216(80.66)	460(7.11)	791(12.23)	0.001
	Yes	1066(14.05)	819(76.83)	78(7.32)	169(18.85)	
**Abnormal CH**	No	6980(92.01)	5599(80.21)	481(6.89)	900(12.89)	0.001
	Yes	553(7.29)	436(78.84)	57(10.31)	60(10.85)	
**LDL/HDL ratio**	Mean (SD)	-	2.23(0.68)	2.40(0.69)	2.54(0.76)	<0.001
	Frequency	-	6033	538	959	
**CH/HDL ratio**	Mean (SD)	-	4.06(1.09)	4.28(1.08)	4.54(1.21)	<0.001
	Frequency	-	6034	538	960	

**Fig. 1:**
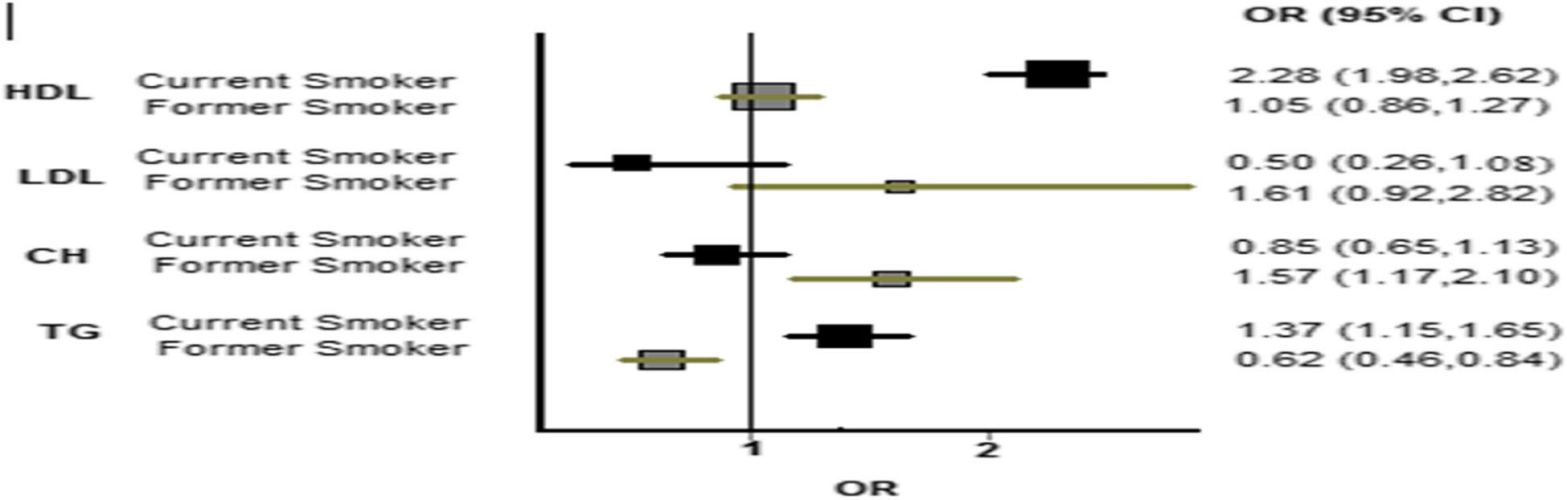
Forest plot of ORs (95% CIs) in smoking cigarette for dyslipidemia status

In addition, current smokers were at significantly greater risk of having abnormal HDL-C [OR (95% CI), 2.28(1.98 -2.62)] and triglycerides [OR (95% CI), 1.37(1.15 -1.65)] than non-smokers. Former smokers had higher risk of having abnormal total cholesterol [OR (95% CI), 1.57(1.17 -2.10)] but the risk of having abnormal HDL-C or LDL-C did not reach the significant level. Furthermore, in former smokers, the risk of having abnormal triglyceride was significantly lower than non-smokers [OR (95% CI), 0.62(0.46 -0.84)](Fig. 1).

**Fig. 2:**
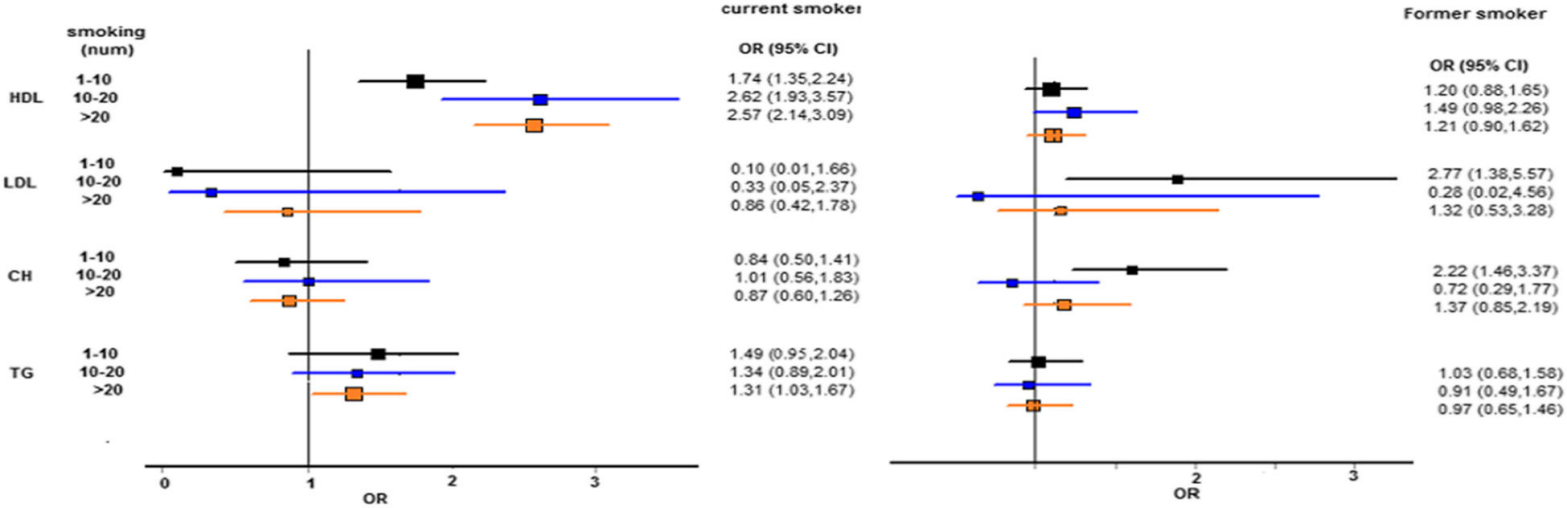
Forest plot of ORs (95% CIs) in number of smoking cigarette using for dyslipidemia status (baseline category: no smoking)

As for the dose-response relationship between the number of smoked cigarettes and abnormal levels of blood lipids, current smokers showed significant abnormal HDL-C and triglyceride levels but such association did not reach the significant level regarding abnormal LDL-C and total cholesterol levels. That is, in those who smoked 1-10, 10-20, and +20 cigarettes, the risk of having abnormal HDL-C was 1.74, 2.62, and 2.57 times higher compared to non-smokers. In addition, triglyceride levels in the current smokers with +20 cigarettes was significantly higher than non-smokers (OR=1.31). However, the number of smoked cigarettes did not draw a significant distinction between the current smokers and non-smokers in terms of LDL-C and total cholesterol levels (Fig. 2).

The chance of developing abnormal HDL-C was strongly correlated with the number of smoked cigarettes, gender (male), higher BMI, and low physical activity. While, age and wealth index were not significant for abnormal HDL-C; age, BMI, and wealth index were closely linked with the total cholesterol level. In addition, the number of smoked cigarettes, BMI, physical activity, and HEI were significant variables for triglyceride levels and they were entered into the relevant logistics model.

For the former smoker group, gender, the number of smoked cigarettes, BMI, and physical activity were significant variables for HDL-C levels. On the other hand, LDL-C levels were correlated with the number of smoked cigarettes, age, and wealth index (without dose-response relationship). Total cholesterol levels were also related to the number of smoked cigarettes (without dose-response relationship), age, BMI, and wealth index. And finally, gender, BMI, physical activity, and nutrition were significant variables for triglyceride levels. Therefore, these variables were entered the relevant model (Table 2).

**Table 2:** multiple logistic regressions for evaluation of association between dyslipidemia items and smoking by adjusted important predictors

Model for	Variables		HDL^a^ OR (95%CI)	LDL^b^ OR (95%CI)	CH^c^ OR (95%CI)	TG^d^ OR (95%CI)
**Current Smoker**	**Gender** (ref: male)	Female	0.28(0.24,0.32)	-	-	-
	**Number of cigarette per day** (ref: non-smoker)	1-10	1.10(0.84,1.43)	1.00(0.98,1.20)	0.88(0.52,1.48)	1.54(1.12,2.13)
		10-20	1.81(1.31,2.51)	0.29(0.041,2.14)	1.06(0.58,1.94)	1.57(1.03,2.38)
		>20	1.87(1.53,2.29)	0.79(0.38,1.64)	0.87(0.59,1.28)	1.69(1.32,2.18)
	**Age group** (ref:35-45)	46-55	-	1.13(0.74,1.74)	1.54(1.23,1.93)	-
		56-65	-	1.88(1.18,2.99)	2.37(1.81,3.09)	-
	**BMI** kg/m^2^ (ref:>18.5)	18.6-24.9	2.19(1.31,3.67)	-	2.99(0.93,9.61)	3.91(1.62,9.50)
		25-29.9	4.85(2.91,8.01)	-	4.99(1.56,6.84)	8.71(4.85,6.26)
		30-34.9	4.89(2.91,8.24)	-	4.89(1.52,7.77)	9.04(5.02,12.48)
		>35	5.09(2.90,8.92)	-	5.14(1.53,8.29)	7.01(3.68,10.05)
	**Physical activity** METs-*hours per week* (ref: 24-36.5)	36.6-44.9(middle)	0.9(0.79,1.04)	-	-	0.75(0.64,0.87)
		≥45(active)	0.71(0.61,0.82)	-	-	0.76(0.62,0.92)
	**Wealth index** (ref: 1st quintile the poorest**)**	2nd	-	0.64(0.37,1.09)	0.81(0.62,1.06)	-
		3rd	-	0.71(0.42,1.19)	0.65(0.49,0.87)	-
		4th	-	0.65(0.38,1.12)	0.63(0.46,0.84)	-
		5th quintile (the richest)		0.47(0.26,0.87)	0.53(0.37,0.77)	-
	**HEI** (ref:1st quintile)	2nd		-	-	0.84(0.68,1.04)
		3rd		-	-	0.79(0.64,0.98)
		4th		-	-	0.76(0.61,0.93)
		5th quintile (the healthy)		-	-	0.85(0.68,1.06)
**Former Smoker**	**Gender** (ref: male)	Female	0.29(0.26,0.33)	-	-	0.47(0.41,0.53)
	**Number of cigarette per day** (ref: non- smoker)	1-10	0.85(0.61,1.18)	2.45(1.21,4.97)	1.95(1.27,3.02)	0.97(0.70,1.35)
		10-20	0.95(0.36,1.46)	1.01(0.69,2.39)	0.65(0.26,1.62)	0.79(0.5,1.26)
		>20	0.71(0.52,0.97)	1.18(0.47,2.97)	1.12(0.68,1.84)	0.86(0.62,1.72)
	**Age group** (ref: 35-45)	46-55	-	1.23(0.79,1.90)	1.62(1.28,2.02)	-
		56-65	-	2.07(1.30,3.31)	2.27(1.75,2.94)	-
	**BMI** kg/m^2^ (ref : ≤18.5)	18.6-24.9	2.98(1.36,6.56)	-	3.15(0.76,13.05)	1.87(0.11,3.85)
		25-29.9	6.22(2.84,3.59)	-	5.33(1.29,21.92)	2.86(0.88,4.83)
		30-34.9	6.29(2.86,13.84)	-	5.10(1.23,21.11)	2.99(1.02,4.97)
		>35	0.89(0.77,1.03)	-	5.37(1.44,21.86)	2.94(0.94,4.93)
	**Physical activity** METs-*hours per week* (ref: 24-36.5)	36.6-44.9 (middle)	0.69(0.59,0.82)	-	-	0.82(0.71,0.93)
		≥45 (active)	0.29(0.26,0.33)	-	-	0.68(0.58,0.81)
	**Wealth index** (ref: 1st quintile the poorest**)**	2nd quintile	-	0.49(0.27,0.89)	0.78(0.59,1.02)	-
		3rd quintile		0.76(0.45,1.29)	0.59(0.44,0.79)	-
		4th quintile		0.77(0.45,1.31)	0.58(0.43,0.78)	-
		5th quintile (the richest)		0.46(0.25,0.85)	0.39(0.29,0.55)	-
	**HEI** (ref:1st quintile)	2^nd^ quintile		-	-	0.85(0.68,1.06)
		3^rd^ quintile		-	-	0.80(0.64,1.01)
		4^th^ quintile	-	-	-	0.77(0.62,0.96)
		5th quintile (the healthy)	-	-	-	0.84(0.66,1.06)

^a^ HDL cut point: 40

^b^ LDL cut point: 160

^c^ CH cut point: 240

^d^TG cut point: 200

For the relationship between the number of smoked cigarettes and blood lipids, the adjusted logistic regression model also showed a significant relationship between the number of cigarettes smoked and HDL-C and triglyceride levels; i.e. with an increase in the number of cigarettes, the risk of having abnormal HDL-C and triglyceride levels increased. In former smokers, as compared to non-smokers, HDL-C, LDL-C, and total cholesterol was significantly correlated with the number of smoked cigarettes. It was observed that the risk of having abnormal HDL-C decreased significantly in cases with +20 cigarettes. Those who smoked 10 cigarettes had significantly higher risk of having abnormal total cholesterol and LDL-C levels than non-smokers. They also showed greater risk of having abnormal total cholesterol and LDL-C levels than subjects who used to smoke +10 cigarettes.

## Discussion

We found a prevalence of 40% for dyslipidemia which was similar to the results of other studies reported in the literature (varying between 14% and 79% ) [27, 28]. Our study was designed to examine the relationship between dyslipidemia and cigarette smoking within a cohort study. For the purpose of this study, we excluded dyslipidemic patients with diabetes and those who were on medication and therefore cannot be generalized to the general population. However, the prevalence of smoking in this study is consistent with the meta-analysis conducted in 2013 [22]. In general, the prevalence of smoking among those aged 35 to 65 was about 14%; nearly 20% in men and less than 2% in women. The results indicate a significant correlation between smoking and blood lipid levels which is not in line with the findings of a similar study in China [14] possibly caused by different populations of these two studies in terms of age and sex structure.

While some studies have shown that smoking reduces total cholesterol, LDL-C and HDL-C with an increase in triglyceride level [29, 30], others have reported that smoking increases total cholesterol, LDL-C, and triglyceride with a decrease in HDL-C level [31]. This contrast was also observed even after controlling the potential confounders (age, sex, and BMI) [5]. This can be, at least, partly due to the association between serum lipids level and other factors including the use of alcohol and hookah (water pipes used to smoke specially made tobacco with the same health risks as cigarette smoking) [13].

Based on multiple logistic regressions, the risk of having abnormal HDL-C in current smokers who smoked at least 10 cigarettes in a month and the risk of having abnormal triglyceride in those who smoked at least 20 cigarettes in a month were significantly higher than non-smokers which were consistent with the results from elsewhere [30–32]. In addition, similar to findings reported in literature, LDL-C and total cholesterol levels in former smokers who used to smoke less than 10 cigarettes in a month were shown to be significantly higher than their non-smoker counterparts [31].

For former smokers, the model showed that the risk of having abnormal HDL-C, LDL-C, and total cholesterol levels was associated with the number of cigarettes smoked. Thus, the risk of having abnormal HDL-C in participants who smoked more than 20 cigarettes was significantly lower than non-smokers. However, former smokers with a history of fewer than 10 cigarettes had a significantly more abnormal total cholesterol levels than non-smokers.

Participants who used to smoke more than 10 cigarettes showed more abnormality in terms of LDL-C and total cholesterol levels compared to subjects who used to smoke a smaller number of cigarettes. Besides, their risk of having abnormal HDL-C (per cigarette) was lower than non-smokers. One assumption was that a higher number of smoked cigarettes may have helped former smokers to have their total cholesterol and HDL-C levels normalized or, they might have opted for a healthier lifestyle through exercise or other measures which were not examined in the present study. More research is required to shed light on the matter.

As for the limitations of the study, its cross-sectional design did not allow for a conclusion about the direction of casualty between smoking and dyslipidemia. Another limitation is regarding the possibility of recall bias regarding the data on smoking as they are self-reported.

## Conclusions

As shown in the present research, current smokers had lower HDL-C but significantly higher triglyceride levels than non-smokers. Former smokers were proved to have a significantly higher total cholesterol levels than non-smokers. Their triglyceride levels also showed great abnormality despite being lower compared to the non-smoker group. The adjustment of confounding variables demonstrated that the risk of having abnormal HDL-C and triglyceride levels in current smokers increased with increase in the number of smoked cigarettes. Yet, after quitting, former smokers (regardless of the number of smoked cigarettes) experienced a more normal HDL-C level than non-smokers. Also, after quitting, the subjects who used to smoke a larger number of cigarettes had a more normal LDL-C and total cholesterol level in comparison to those who used to smoke less. Stricter measures including prohibiting smoking in public places or increasing taxes on tobacco are recommended to reduce the burden of CVDs either directly or through cigarette smoking in the community.

## Data Availability

The datasets used and/or analyzed during the current study are available from the corresponding author on reasonable request.

## Abbreviations

RaNCD: Ravansar non-communicable disease
PERSIAN: Prospective epidemiological research studies in Iran
mg/dL: Milligrams per deciliter
NHIS: National health insurance scheme
SES: Socio-economic status
PCA: Principal component analysis
HEI: Healthy eating index
LDL: Low-densitylipoprotein
HDL: High-density lipoprotein
TG: Triglyceride
CH: Cholesterol
